# False-positive *Aspergillus* galactomannan immunoassay in the glucose component of total parenteral nutrition products

**DOI:** 10.1128/spectrum.01673-23

**Published:** 2023-10-06

**Authors:** Eunbin Chong, Jae-Hoon Ko, Doo Ri Kim, Young Ho Lee, Jinyoung Yang, Haein Kim, Kyungmin Huh, Cheol-In Kang, Doo Ryeon Chung, Kyong Ran Peck, In Hwa Jeong, Tae Yeul Kim, Hee Jae Huh, Nam Yong Lee, Areum Shin, Yae-Jean Kim, You Min Sohn, Sun Young Cho, Eun-Suk Kang

**Affiliations:** 1 Department of Laboratory Medicine and Genetics, Samsung Medical Center, Sungkyunkwan University School of Medicine, Seoul, South Korea; 2 Division of Infectious Diseases, Department of Medicine, Samsung Medical Center, Sungkyunkwan University School of Medicine, Seoul, South Korea; 3 Division of Infectious Diseases and Immunodeficiency, Department of Pediatrics, Samsung Medical Center, School of Medicine, Sungkyunkwan University, Seoul, South Korea; 4 Department of Pharmaceutical Services, Samsung Medical Center, Sungkyunkwan University School of Medicine, Seoul, South Korea; Quest Diagnostics, San Juan Capistrano, California, USA

**Keywords:** *Aspergillus*, galactomannan, false-positive, total parenteral nutrition, glucose monohydrate

## Abstract

In October 2022, we experienced a significant increase in samples showing high galactomannan (GM) indexes ranging from 6.22 to 10.58, as determined by the Platelia *Aspergillus* antigen immunoassay, also known as the GM test. After reviewing the medical records of nine GM antigenemia cases that did not show evidence of invasive aspergillosis, we found that these patients had received total parenteral nutrition (TPN) products from the same manufacturer, whose supplier of the glucose component had recently changed. The TPN products supplied by the specific manufacturer in October were subjected to a GM assay. The glucose component of the products from three different lot numbers exhibited strong positive results in the GM assay. Microbiological investigations through fungal culture and PCR on the TPN products were negative. The present study demonstrated that the glucose component of the TPN products contained a high level of GM antigen, which caused false-positive GM test results. The source of GM in the glucose component was glucoamylase, which was produced from *Aspergillus niger* to obtain glucose monohydrate from starch. Investigation of three commercially available glucoamylase products exhibited positive GM and 1,3-β-D-glucan tests with various titers positive up to 1:1,000 dilutions, while fungal cultures were all negative. Quality assurance measures of TPN products to prevent GM contamination should be emphasized during the manufacturing process to avoid unnecessary additional diagnostic procedures and overtreatment of invasive aspergillosis due to false-positive GM tests.

**IMPORTANCE:**

This manuscript describes an occurrence of false-positive GM tests in patients receiving TPN products from a manufacturer who had recently changed the supplier of the glucose component. We describe the clinical presentation of nine false-positive cases and the results of serologic and microbiological investigations of the TPN products suspected of contamination with GM. Attempts to detect GM in parenteral nutrition products were made since the detection of GM in sodium gluconate-containing solutions in 2007, but none of them identified the source of elevated GM indexes in TPN products. However, the present study demonstrated that the glucose component of the TPN products contained a high level of GM antigen, which caused false-positive GM assay results. The source of GM was glucoamylase, which was derived from *A. niger* in the manufacturing process. Physicians and clinical microbiology laboratories should be aware of this issue to improve interpretation and patient care.

## OBSERVATION

Platelia *Aspergillus* antigen immunoassay (Bio-Rad, CA, USA), also known as the galactomannan (GM) test, is a useful diagnostic method to detect GM from serum or bronchoalveolar lavage fluid for an early diagnosis of invasive pulmonary aspergillosis (IPA) (1, 2). Various factors have been reported to cause the false-positive GM test, such as underlying disease, antibiotics, and sodium gluconate-containing solutions (3 – 8). In October 2022, we experienced nine false-positive cases with a high GM index (GMI) ranging from 6.22 to 10.58. We discovered that the false-positive GM assay occurred in patients receiving total parenteral nutrition (TPN) products from the same manufacturer, whose supplier of glucose component had recently changed (lot number a-e of Table 1). Herein, we present the clinical presentation of false-positive cases and the results of serologic and microbiological investigations of TPN products with glucose component suspected of contamination with GM.

**TABLE 1:** Platelia *Aspergillus* antigen immunoassay, fungal culture, and PCR results of TPN products^*a*^ ^,*b*^

Product	Lot no.	Chamber	Dilution	GMI	Fungal culture	*Aspergillus* PCR
1 L product	a	Glucose	None	NA	NA	Not detected
		Amino acids	None	NA	NA	NA
		Lipid	None	NA	NA	NA
		Total mixture	None	0.43 (negative)	NA	NA
	b	Glucose	None	NA	NA	Not detected
		Amino acids	None	NA	NA	NA
		Lipid	None	NA	NA	NA
		Total mixture	None	0.14 (negative)	NA	NA
	c	Glucose	None	NA	NA	Not detected
			1:3	6.65 (positive)	NA	NA
			1:10	6.83 (positive)	NA	NA
		Amino acids	None	0.21 (negative)	NA	NA
		Lipid	None	0.16 (negative)	NA	NA
		Total mixture	None	6.70 (positive)	NA	NA
1.5 L product	d	Glucose	None	3.09 (positive)	No growth	Not detected
			1:3	10.06 (positive)	NA	NA
			1:10	10.22 (positive)	NA	NA
		Amino acids	None	0.30 (negative)	No growth	NA
		Lipid	None	0.16 (negative)	No growth	NA
		Total mixture	None	9.61 (positive)	NA	NA
	e	Glucose	None	3.27 (positive)	No growth	Not detected
			1:3	10.00 (positive)	NA	NA
			1:10	11.18 (positive)	NA	NA
		Amino acids	None	0.30 (negative)	No growth	NA
		Lipid	None	0.16 (negative)	No growth	NA
		Total mixture	None	10.34 (positive)	NA	NA

^*a*^ The positive result is defined as GMI > 0.55, the negative result as GMI < 0.45, and the equivocal result as GMI 0.45–0.55.

^*b*^ Abbreviations: GMI, galactomannan index; NA, not available; PCR, polymerase chain reaction; TPN, total parenteral nutrition.

**Fig 1:**
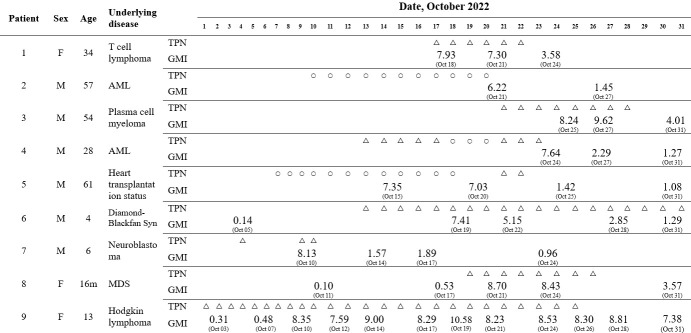
Characteristics of nine patients with timeline of TPN administration and Platelia *Aspergillus* antigen immunoassay results. Circles represent prescription of 1.5 L TPN product, and triangles represent prescription of 1 L TPN product. The positive result is defined as GMI > 0.55, the negative result as GMI < 0.45 and the equivocal result as GMI 0.45–0.55. Abbreviations: AML, acute myeloid leukemia; GMI, galactomannan index; MDS, myelodysplastic syndrome; Syn, syndrome; TPN, total parenteral nutrition.

In October 2022, our hospital encountered nine patients who had high GMI but no evidence of IPA. They were immunocompromised and subjected to GM test for screening to detect the early phase of IPA prior to the development of clinical signs. Their CT scans did not show any radiological abnormalities, and microbial cultures on their blood, urine, and sputum yielded negative results. Figure 1 shows the characteristics of the nine patients, the timeline of TPN administration, and their GM assay results. All patients were severely immunocompromised, with five recently receiving peripheral blood stem cell transplantations (patients 1, 3, 6, 7, and 9), two undergoing intensive cytotoxic chemotherapy (patients 2 and 4), and two taking immunosuppressive drugs (patients 5 and 8). After reviewing the patients’ medical records, we discovered that they had all received TPN from the same manufacturer. Patients 2, 4, and 5 received the 1.5 L TPN product (lot number d or e), while the others received the 1 L product (lot number a, b, or c). In follow-up tests, serum GMI decreased by more than half in most patients, within an average of 4 days after discontinuing TPN infusion, except for patients 6 and 9. In patient 6, serum GMI decreased even though TPN was administered continuously. The decrease in GMI may be due to the change in the lot number of TPN from contaminated to non-contaminated products (exact lot number could not be confirmed) since patient 6 was transferred to a different ward around this time. In patient 9, serum GMI remained persistently high for some time even after stopping TPN infusion, which seems to be related to the prolonged period of TPN administration (25 days).

The Platelia *Aspergillus* antigen immunoassay (Bio-Rad, CA, USA) was performed on the TPN solutions supplied by the specific manufacturer in October 2022 according to the manufacturer’s instructions. The results were summarized in Table 1. Each TPN product has three chambers within the bag for the separate components. Samples for GM tests were obtained from each chamber containing glucose, amino acid, and lipid and whole mixtures of the three compartments using an aseptic procedure. Total mixtures of the three components were tested as an initial screen to detect lot numbers causing high GMI in patients. The mixtures of lot numbers c, d, and e, which included glucose provided by newly changed supplier, revealed positive GM test results with GMI of 6.70, 9.61, and 10.34, respectively. The mixture of lot numbers a and b had glucose provided by the previous supplier, and their GM tests were negative. Additionally, when testing each compartment individually, amino acid and lipid components of lot numbers c, d, and e were negative. Glucose components of lot numbers c, d, and e were diluted with negative sera at 1:3 and 1:10 and showed high GMI ranging from 6.65 to 11.18, while undiluted glucose samples displayed relatively low GMI around 3.0. This phenomenon probably indicates the presence of a post-zone effect due to excess GM antigens in the glucose compartments (9).

Microbial cultures were performed on the TPN solutions to detect viable fungal microorganisms. A total of six fungal cultures were performed with undiluted solutions from each chamber with lot numbers d and e(Table 1). Cultures were incubated on Sabouraud dextrose agar plates for 2 weeks, but no growth of any microorganisms was observed. To investigate the presence of *Aspergillus* DNA fragments in the TPN solution, PCR was performed on samples from the glucose compartments with lot numbers a–e(Table 1). The presence of *Aspergillus* DNA was tested using nested PCR with the outer primer set AFU7S and AFU7AS, and the inner primer set AFU5S and AFU5AS, as described previously (10). No *Aspergillus* DNA was detected in the TPN solutions that previously showed positive GM tests (Fig. S1).

Once we reported the potential contamination of TPN products to the manufacturer based on our initial investigation, they conducted an internal assessment and similarly found positive GM test results in glucose components manufactured with raw materials from the new supplier but not in glucose components from the previous supplier. The manufacturer began using glucose monohydrate from the new supplier to produce the glucose component since July 2022. The new supplier was using glucoamylase derived from an industrial fermentation process from *Aspergillus niger* to obtain glucose monohydrate (according to communication with the manufacturer). *A. niger*, which is generally recognized as safe, has been widely used for the production of commercial glucoamylase (11). GM generated from *A. niger* was present in the glucose component of TPN products, resulting in false-positive results. Since the TPN products from the former supplier of glucose monohydrate did not show GM positivity, it was likely that they used a different microorganism such as *Bacillus* species, which is also known to be used to produce glucoamylase (12) or had a refined process to remove or reduce GM, but they did not provide further information. To investigate if glucoamylase was the source of the GM found in the glucose component, we purchased three commercially available glucoamylase products derived from *A. niger*. All of them tested positive for GM and 1,3-β-D-glucan with varying intensity positive up to 1:1,000 dilutions, according to the manufacturers, and showed negative fungal culture (Table S1). This finding supported that the GM antigen found in the glucose sample was originated from glucoamylase.

In previous publications, false-positive GM tests have been reported with the use of piperacillin-tazobactam (Pfizer, NY, USA) (4, 5, 13, 14), Plasma-Lyte (Baxter, IL, USA) (7, 8, 15), and NP2 Enfant AP-HP parenteral nutrition (Fresenius, France) (6). It has been suggested that the detection of GM in antibiotics originated from the cell wall of *Penicillum* species, which were used to produce semisynthetic drugs like piperacillin-tazobactam (16, 17). Similarly, GM in Plasma-Lyte and NP2 parenteral nutrition was generated from *A. niger* during the industrial fermentation process of sodium gluconate (6, 15). The GM carryover of both piperacillin-tazobactam and sodium gluconate containing solutions was resolved after refinement of the production process (18, 19). Since the detection of GM in sodium gluconate containing intravenous solutions in 2007, attempts to identify the presence of GM in other parenteral nutrition products have been made. Two *in vitro* studies tested 19 intravenous solutions that are used to prepare TPN and four different TPN products, respectively (20, 21). In those studies, no measurable GMI were observed in the tested TPN solutions, suggesting that GM contamination of TPN product does not occur frequently.

Although false-positive cases in the present study were not associated with direct fungal contamination, the false-positive GM results hinder optimal management of immunocompromised patients. They cause unnecessary additional diagnostic procedures and treatment, exposing patients to radiation from CT scans to identify evidence of IPA, adverse drug events from antifungal agents, and extra medical expenses from needless tests and treatment. Quality assurance measures of TPN products preventing GM contamination should be emphasized during the manufacturing process. Physicians and clinical microbiology laboratories should be aware of this issue to improve interpretation and patient care.

## SUPPLEMENTAL MATERIAL

Supplemental material spectrum.01673-23-s0001.docx Fig. S1 and Table S1.

ASM does not own the copyrights to Supplemental Material that may be linked to, or accessed through, an article. The authors have granted ASM a non-exclusive, world-wide license to publish the Supplemental Material files. Please contact the corresponding author directly for reuse.
