# Supplementary material for: False-positive Aspergillus galactomannan immunoassay in the glucose component of total parenteral nutrition products
Source: Microbiol Spectr. 2023 Oct 6;11(6):e01673-23. doi: 10.1128/spectrum.01673-23 (PMC10715174; doi:10.1128/spectrum.01673-23)
Supplement: Supplemental material — Fig. S1 and Table S1. [file spectrum.01673-23-s0001.docx]

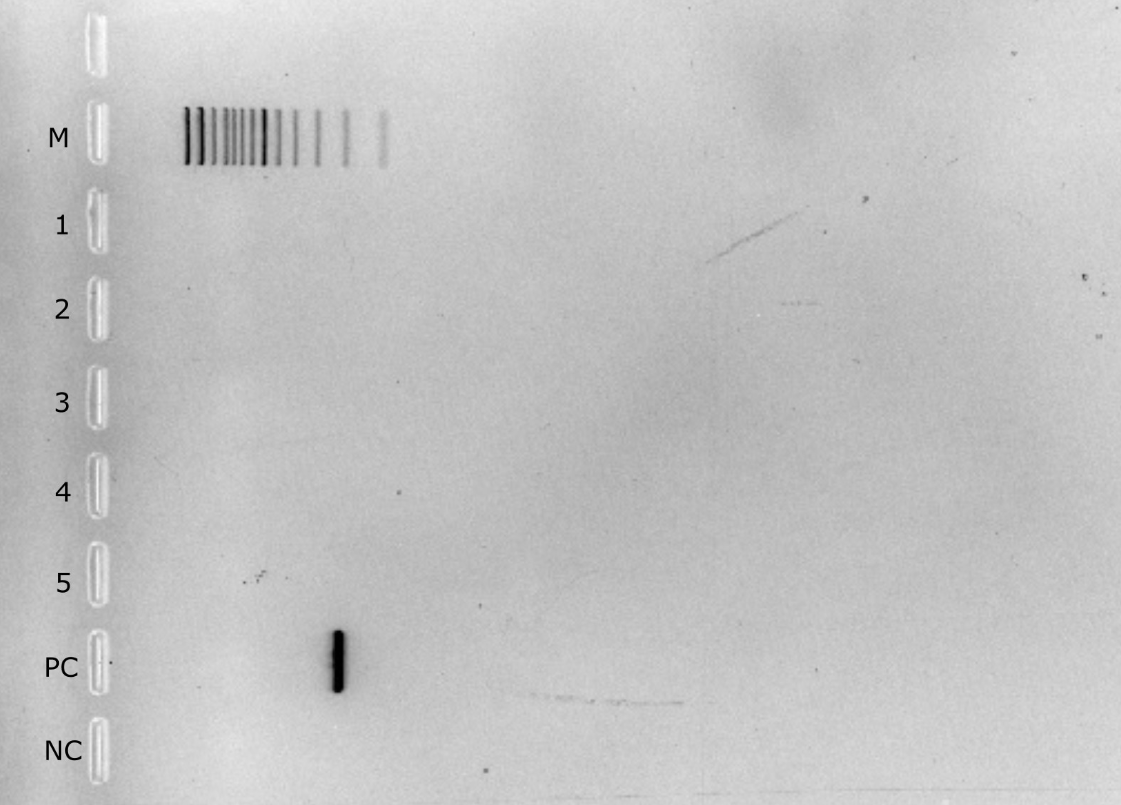


**Fig. S1.** *Aspergillus* DNA PCR test results of TPN products

Lane 1-5 are glucose samples with lot numbers a-e, respectively. Lane M represents DNA marker (100bp ladder). DNA extracted from *Aspergillus niger* was used as a positive control (236bp) and distilled water was used as a negative control. All the tests of TPN products were negative.

Abbreviation: PC, positive control; NC, negative control

**Table S1.** Platelia *Aspergillus* antigen immunoassay, 1,3-β-D-glucan test, and fungal culture of commercially available glucoamylase products

| **Glucoamylase**  **manufacturer** | **Dilution** | **GMI** | **BDG, pg/mL** | **Fungal culture** |
| --- | --- | --- | --- | --- |
| S | None | **5.61 (positive)** | **672.9 (positive)** | No growth |
|  | 1:10 | **1.90 (positive)** | NA | NA |
|  | 1:100 | 0.19 (negative) | NA | NA |
|  | 1:1,000 | 0.11 (negative) | NA | NA |
|  | 1:10,000 | 0.05 (negative) | NA | NA |
| R | None | **0.60 (positive)** | **914.2 (positive)** | No growth |
|  | 1:10 | 0.33 (negative) | NA | NA |
|  | 1:100 | 0.04 (negative) | NA | NA |
|  | 1:1,000 | 0.04 (negative) | NA | NA |
|  | 1:10,000 | 0.10 (negative) | NA | NA |
| N | None | **7.43 (positive)** | **> 1,000 (positive)** | No growth |
|  | 1:10 | **7.33 (positive)** | **672.9 (positive)** | NA |
|  | 1:100 | **7.33 (positive)** | **> 1,000 (positive)** | NA |
|  | 1:1,000 | **3.49 (positive)** | **197.6 (positive)** | NA |
|  | 1:10,000 | 0.29 (negative) | < 10.0 (negative) | NA |

Phosphate buffered saline was used for the dilution of the glucoamylase products for Platelia *Aspergillus* antigen immunoassay and control serum from a healthy donor was used for 1,3-β-D-glucan test.

The positive results are defined as GMI > 0.55 and BDG ≥ 80.0 pg/mL, the negative results as GMI < 0.45 and BDG < 60.0 pg/mL, and the equivocal results as GMI 0.45–0.55 and BDG 60.0–79.9 pg/mL

Abbreviations: GMI, galactomannan index; BDG, 1,3-β-D-glucan; NA, not available
